# RNA-Seq-Based Molecular Classification Analyses in Colorectal Cancer and Synchronous Adenoma

**DOI:** 10.3390/cancers15194851

**Published:** 2023-10-04

**Authors:** Ji Won Choi, Gi-Young Lee, Sangsoo Kim, Kwangsung Ahn, In-Gu Do, Kyung-Uk Jung, Hyung-Ook Kim, Hungdai Kim, Dong-Il Park, Soo-kyung Park

**Affiliations:** 1 Department of Biological Sciences, Sungkyunkwan University, Suwon 16419, Republic of Korea; cjw9588@skku.edu; 2 Functional Genome Institute, PDXen Biosystems Co., Daejeon 34027, Republic of Korea; 3 Department of Bioinformatics and Life Science, Soongsil University, Seoul 06978, Republic of Korea; gy48085@gmail.com (G.-Y.L.);; 4 Department of Pathology, Kangbuk Samsung Hospital, School of Medicine, Sungkyunkwan University, Seoul 03181, Republic of Korea; ingu.do@samsung.com; 5 Department of Surgery, Kangbuk Samsung Hospital, School of Medicine, Sungkyunkwan University, Seoul 03181, Republic of Koreaho115.kim@samsung.com (H.-O.K.);; 6 Division of Gastroenterology, Department of Internal Medicine, Kangbuk Samsung Hospital, School of Medicine, Sungkyunkwan University, Seoul 03181, Republic of Korea

**Keywords:** colorectal cancer, colon polyp, adenoma, RNA sequencing

## Abstract

**Simple Summary:**

The study focuses on colorectal cancers (CRC) and their molecular subtypes (CMS) based on gene expression. A revised classification system, iCMS, incorporates epithelial status, microsatellite instability, and fibrosis. The research uses iCMS to investigate the connection between CRC and adenomas, examining gene expression and cell types. An in silico method called CiberSortx estimates cell proportions, with a random forest model classifying CMS classes. Results suggest most CRCs are CMS2 or CMS3, while a novel subtype, iCMS2-F/iCMS3-F, is proposed due to enrichment in myofibroblasts. The study highlights the potential of iCMS and in silico methods for CRC and adenoma analysis.

**Abstract:**

Colorectal cancers (CRC) are classified into consensus molecular subtypes (CMS) based on gene expression profiles. The revised classification system iCMS was proposed by considering intrinsic epithelial status, microsatellite instability (MSI), and fibrosis. This study aimed to provide molecular evidence for the adenoma–carcinoma sequence concept by examining CRC and synchronous adenomas using iCMS. Epithelial CMS cell proportion was estimated using CiberSortx, an in silico cell fractionation method that included CMS cell types among the reference cell types. A random forest (RF) model estimated the posterior probabilities of CMS classes, which were compared with the CiberSortx results. Gene expression profiles of the published iCMS signature panel were retrieved from our dataset and subjected to heatmap clustering for classification. Bulk RNA sequencing data were collected from 29 adenocarcinomas and 11 adenoma samples. CiberSortx showed all CRC contained either CMS2 or CMS3 as the major epithelial cancer cell type. The RF model classified approximately half of the CRC as CMS4, whereas CMS4 was hardly detected by CiberSortx. Because they were enriched with myofibroblasts as per the CiberSortx classification, we tentatively designated them as iCMS2-F/iCMS3-F. iCMS coupled with the application of an in silico cell fractionation method can provide the molecular dissection of CRC and adenoma.

## 1. Introduction

Colorectal cancer (CRC) is the fourth most frequently diagnosed cancer and the second most common cause of cancer-related deaths worldwide [1]. The emergence and development of omics technology have revealed the genetic pathogenesis of CRC at the gene, RNA, and protein levels. Based on gene expression profiles from bulk tumors, CRC is classified into four types called consensus molecular subtypes (CMSs) [2]. CMS1 tumors harbor a microsatellite instability (MSI) with high CpG island methylator phenotypes; CMS2 tumors show dysregulated WNT signaling; CMS3 tumors are characterized by KRAS mutation; CMS4 tumors have TGF-beta activation with poor survival. This classification scheme was developed based on bulk transcriptome data; however, the recent advent of single-cell-level transcriptomics provides a more detailed CRC molecular dissection regarding CMS. Joanito et al. proposed a new classification system by analyzing several CRC single-cell RNA-seq (scRNA-seq) datasets [3]. Based on two intrinsic subtypes, iCMS2 and iCMS3, refined CMSs, defined the intrinsic epithelial axis of CRC and proposed a refined “IMF” classification with five subtypes, combining intrinsic epithelial subtype (I), MSI status (M), and fibrosis (F).

However, adenoma-associated CMSs remain unclear. The adenoma–carcinoma sequence concept in CRC is widely accepted. After the first report in the 1970s [4], an improved understanding of polyp-to-cancer progression was achieved by developing a multi-hit genetic model of CRC carcinogenesis [5]. Diverse genetic and epigenetic changes including chromosomal instability, MSI, and the CpG island methylator phenotype are well-known molecular events of CRC [6,7,8]. However, the relationship between transcriptome characteristics and the adenoma–carcinoma sequence in CRC remains unclear.

Here, we report a bulk transcriptomic study on CRC and synchronous colorectal adenomas using RNA sequencing (RNA-seq). First, each sample was classified using the traditional CMS scheme. The cell-type composition of each sample was inferred using CiberSortx. The CMS classifier, a random forest (RF) machine learning model, was employed to classify each sample into CMS classes. Next, each sample was classified into revised iCMS classes using published panel genes. Finally, variant calling was performed on CRC samples using RNA-seq to profile somatic mutations in tumor-related pathways. These results were corroborated to determine whether the adenoma samples displayed a congruent CMS with their matched adenocarcinoma samples.

## 2. Materials and Methods

### 2.1. Study Samples

We included patients diagnosed with colorectal adenocarcinoma or colorectal adenocarcinoma with synchronous colorectal adenoma between July 2017 and July 2019 at the Kangbuk Samsung Hospital. During this period, all colorectal adenocarcinoma tissues were collected from surgical specimens or endoscopic biopsies. For the transcriptomic study using RNA-seq, we included 29 adenocarcinomas and 11 adenomas from 29 patients because some patients had more than one synchronous adenoma (Table 1). The Institutional Review Board of Kangbuk Samsung Hospital, Korea, approved the study. All participants provided informed consent (KBSMC 2017-05-008-001).

### 2.2. Sample Preparation, Library Construction, and RNA-Seq

Total RNA was extracted with TRIzol™ (Thermo Fisher Scientific, Waltham, MA, USA) from fixed fresh tissue embedded in RNAlater™ Stabilization Solution (Thermo Fisher Scientific). Sequencing experiments were performed at the Korea Research Institute of Bioscience and Biotechnology. The TruSeq^®®^ Stranded Total RNA library prep kit was used for Illumina TruSeq sequencing library construction (Illumina, San Diego, CA, USA).

### 2.3. Mapping Preparation and Clean-Up Reads

RNA-seq analysis was performed using GATK (Genome analysis toolkit) v4.1.9.0 and its best-practice pipelines with minor modifications. Following the GATK somatic short variant discovery (https://gatk.broadinstitute.org/hc/en-us/articles/360035894731-Somatic-short-variant-discovery-SNVs-Indels- (accessed on 25 January 2021)) and RNA-seq short variant discovery pipelines (https://gatk.broadinstitute.org/hc/en-us/articles/360035531192-RNAseq-short-variant-discovery-SNPs-Indels- (accessed on 25 January 2021)), the mapped reads were polished for further analysis.

Mapping reads to the reference genome was completed using STAR v2.7.7a. STAR-2pass mode; the “--twopassMode Basic” option was used to prepare the mapped reads. The reduced GRCh38 version of the sequences (manually discarding ambiguous chromosomes, except for 22 autosomes, chrX, and chrM) was used as a reference genome sequence for further analysis.

After mapping, the duplicate reads were marked using MarkDuplicates. Next, read group information (each run ID of a sequencing instrument) was added to BAM files before base recalibration using AddOrReplaceReadGroup in GATK4. The next step was performed using SplitNCigarReads, an RNA-seq short-variant discovery pipeline for handling RNA-seq reads (split reads as splice junctions) against a reference genome. Finally, base quality score recalibration (BQSR) was performed using BaseRecalibrator and ApplyBQSR. In BQSR, exome variant data from the gnomAD v2.1.1 liftover version were used as known variant sites.

### 2.4. CMS Prediction and CiberSortx

For CMS prediction, the mapped RNA-seq reads were quantified using featureCounts [9] and the raw counts were normalized using the trimmed mean of M-values and converted to counts per million (CPM) using edgeR in R/Bioconductor [10]. We inferred CMS classes for each sample using two different methods.

First, we applied CiberSortx, an in silico cell fraction estimation algorithm, to CPM [11]. Our bulk RNA-seq data represented the composite gene expression of various cell types present in the specimens. As our surgical and biopsy samples were used without dissection or purification to enrich the cancer cells, cancer cell proportion was expected to vary among the samples. One way to delineate the proportion of various cell types in surgical specimens is by the computational cell sorting of bulk gene expression profiles based on cell-type reference profiles that are typically generated from an scRNA-seq dataset of a tissue of interest. Based on the signature expression patterns of several reference cell types, the bulk gene expression was modeled as a linear combination of the reference cell types. We built a reference signature matrix from a Korean CRC scRNA-seq dataset comprising 23 cancer and 10 normal samples (GSE132465). The corresponding gene expression matrix of 63,689 cells was processed using CiberSortx for batch correction in the S-mode and subsequent signature matrix creation. Using the signature matrix and bulk CPM, CiberSortx estimated the fractions of the reference cell types for each sample. Second, we applied the CMS classifier v1.0.0, an RF algorithm available as an R package v4.1.0, directly to the log2-transformed CPM [2]. The algorithm estimates the posterior probabilities of four CMS classes for each sample. It reports the class with the maximum posterior probability.

### 2.5. Variant Calling and Annotation

After the analysis-ready reads were prepared, somatic variant calling was conducted using Mutect2, a tumor-only protocol, following the GATK somatic short-variant discovery pipeline.

Following the GATK pipeline for somatic variant discovery, contamination was calculated and used to filter variants together with known germline variants and common single nucleotide polymorphisms. Finally, the filtered variant was annotated using Funcotator. The somatic variant data were downloaded using FuncotatorDataSourceDownloader. The option “--remove-filtered variants” was applied to eliminate non-passed variants from the output file.

We grouped variations by sample and four tumor-related pathways (WNT, TGFB, TP53and MARK signaling). To calculate the effect of each variant included in the pathway, a CHASM cancer-specific high-throughput annotation of somatic mutation FDR score was evaluated by CRAVAT (Cancer-Related Analysis of Variants Toolkit, https://www.cravat.us/CRAVAT/).

## 3. Results

### 3.1. In Silico Cell Fraction Estimation Using CiberSortx

**Figure 1:**
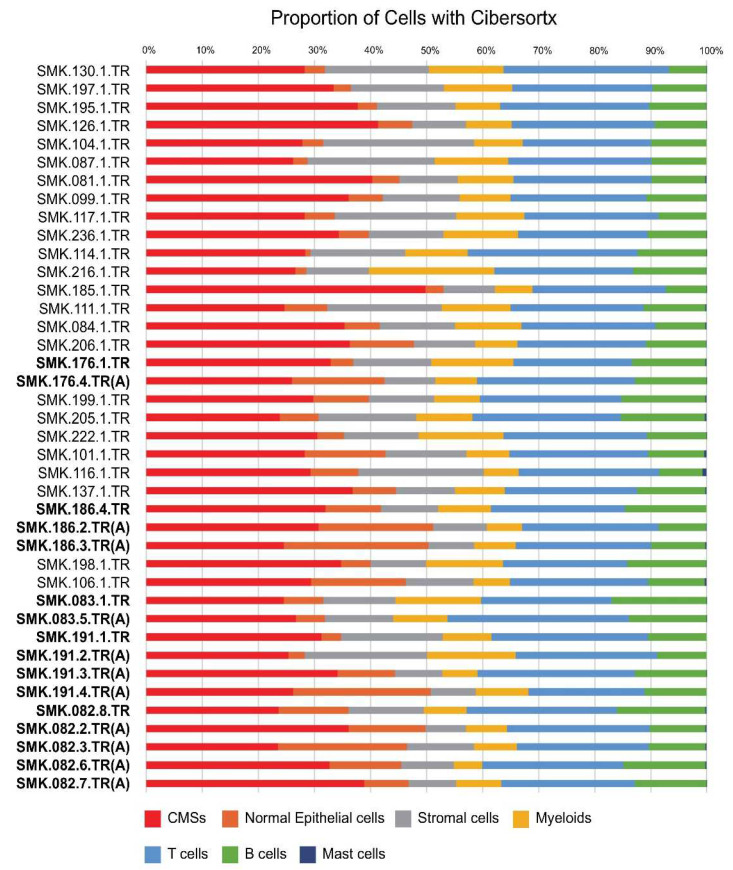
The cellular heterogeneity analyzed by CiberSortx for 29 adenocarcinoma and 11 adenoma samples from 29 CRC patients. Bold point with the same number following SMK refers to the same patient. (A) means adenoma sample, and the rest are cancer samples. Each color shows the proportion of seven major cell types.

A signature matrix from a Korean CRC scRNA-seq dataset represented the expression profiles of 6194 genes in 38 cell types. Using the signature matrix as a reference profile, the in silico inference of the cell-type composition of the samples was performed using CiberSortx. Figure 1 shows the proportions of the seven major cell types (CMSs, normal epithelial, stromal, myeloid, T, B, and mast cells). Among the 29 adenocarcinoma and 11 adenoma samples from the 29 patients, CMSs accounted for approximately 31.1% (SD, 5.8%), followed by T cells, B cells, and myeloid, accounting for approximately 25.1% (SD, 2.3%), 11.2% (SD, 2.4%), and 10.1% (SD, 3.5%), respectively. Normal epithelial and stromal cells together accounted for approximately 22.4% (SD, 5.9%), whereas mast cells were hardly visible.

**Figure 2:**
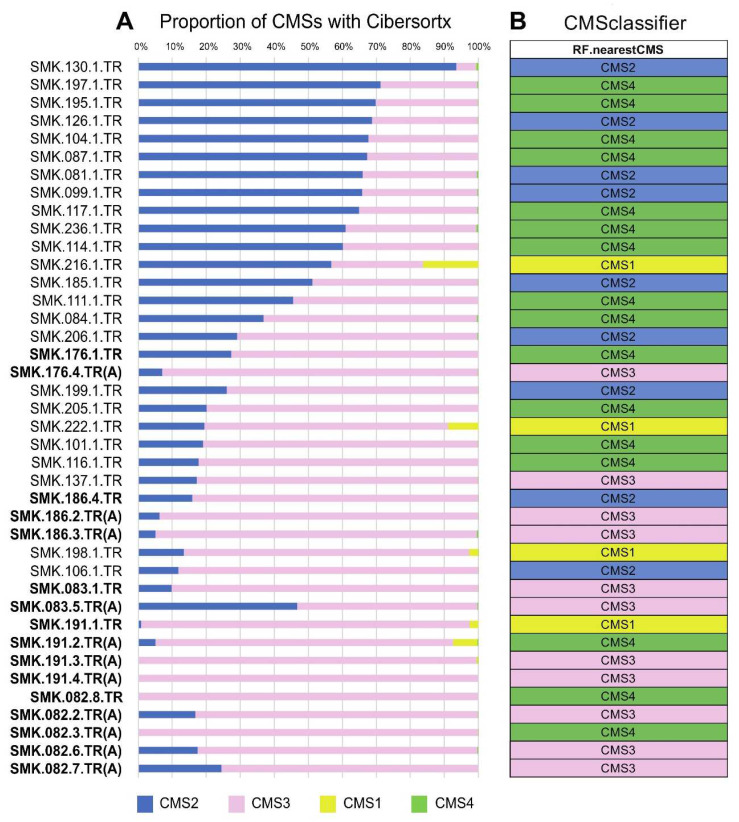
The CMS type predicted by the CiberSortx and CMSClassifier RF model. (**Panel A**) presents stacked bar plots displaying the CMS types, and (**Panel B**) shows the CMS types predicted by the RF model. Bold point with the same number following SMK refers to the same patient. (A) means adenoma sample, and the rest are cancer samples.

Our signature matrix contained four cancer cell types: CMS1, CMS2, CMS3, and CMS4. Figure 2A shows the proportion of cancer cells in each sample. Approximately half of the adenocarcinoma samples contained approximately 50% or more CMS2 cells, whereas in the other adenocarcinoma and adenoma samples, CMS3 was the major type. Among our samples, a few contained noticeable CMS1 cells; CMS4 cells were hardly detected.

The RF classification results are shown in Figure 2B. Approximately half of the samples were classified as CMS4 by these RF tools, disagreeing with the CiberSortx results, where the proportion of CMS4 in all samples was low. Conversely, in samples classified as either CMS2 or CMS3 by the RF classifiers, the cell type was generally the most abundant in the CiberSortx results. The samples with noticeable CMS1 proportions in the CiberSortx results were classified as CMS1 by the RF tools.

### 3.2. Classification by iCMS System

**Figure 3:**
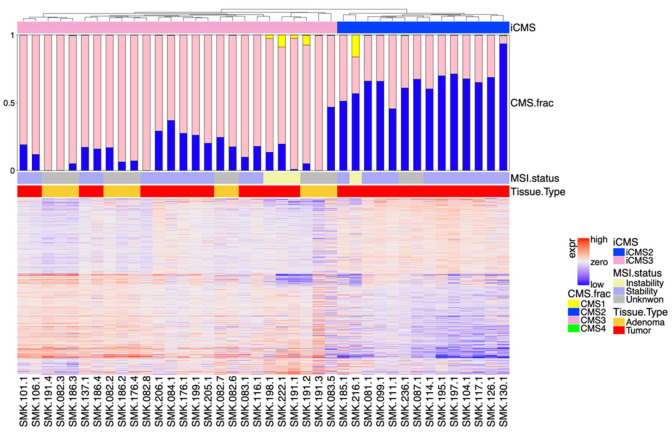
A heatmap using genes reported to classify iCMS2 and iCMS3. 29 adenocarcinoma and 11 adenoma samples were classified to iCMS2 and iCMS3 using a dendrogram. Tissue type and MSI status were annotated above the heatmap, and CMS fractions predicted by CiberSortx were represented as bar plots.

Based on Joanito et al.’s revised CRC classification system using scRNA-seq datasets, we reanalyzed the bulk transcriptome. Of the 715 genes reported as a signature panel for classifying iCMS2 vs. iCMS3 [3], 674 were present in our filtered dataset. Heatmap clustering of these genes is shown in Figure 3. Our CiberSortx results are consistent with the iCMS classification. All CRC samples that had been classified as CMS2 by CiberSortx (*n* = 13) were classified as iCMS2, and most of the CRC samples classified as CMS3 by CiberSortx (*n* = 16) were classified as iCMS3 (*n* = 15, 93.7%). There were a few CMS3 major cases with a noticeable but small proportion of CMS1. Because these samples were of the MSI type, we classified them as iCMS3-MSI. While the CiberSortx results were consistent with the iCMS system, the RF classification results were discordant with the iCMS classification. Our iCMS2 cluster (*n* = 14) comprised samples originally classified as CMS2 (*n* = 5), CMS4 (*n* = 8), and CMS1 (*n* = 1) by RF classification. For the samples in the iCMS3 cluster (*n* = 15), the major types predicted by RF were CMS2 (*n* = 4), CMS4 (*n* = 6), and CMS1 (*n* = 2).

**Figure 4:**
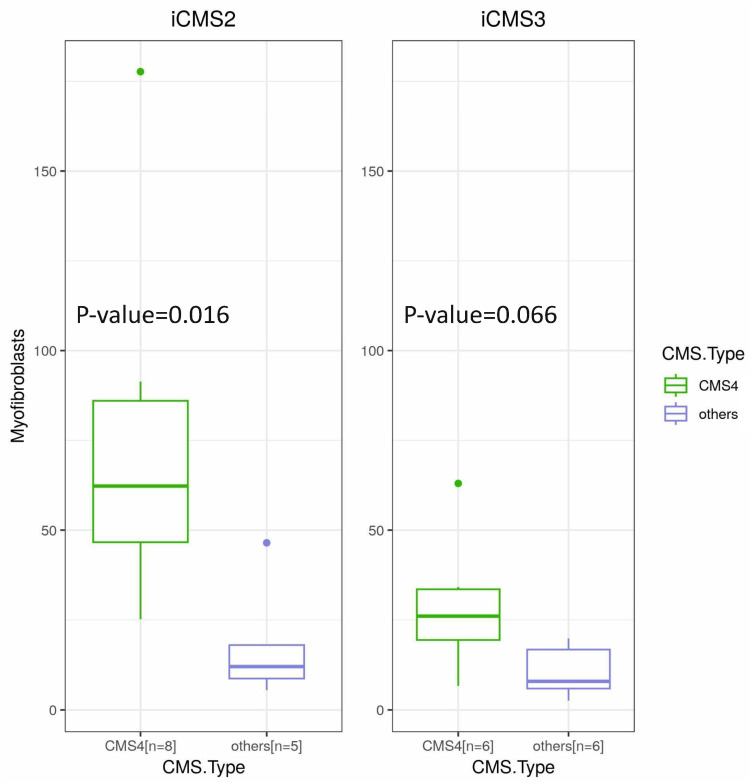
The proportion of myofibroblasts in the samples classified as CMS4 and others (CMS2, CMS3) by the RF model. The samples predicted as CMS4 by the RF model showed pronounced proportion of myofibroblasts in both groups that were classified to iCMS2 (**left**) (*t*-test, *p* = 0.016) and iCMS3 (**right**) (*t*-test, *p* = 0.066) and thus assigned as iCMS2-F and iCMS3-F, respectively. The *y*-axis represents the myofibroblast proportions multiplied by 1000 for visualization.

RF classified approximately half of the CRC samples as CMS4, and all these samples were classified as either iCMS2 or iCMS3 by CiberSortx. The samples that were classified as CMS4 by RF and assigned to iCMS2 or iCMS3 consistently had higher proportions of myofibroblasts than their counterparts in iCMS2 (*t*-test, *p* = 0.016) or iCMS3 (*t*-test, *p* = 0.066) (Figure 4). Therefore, we tentatively classified them as iCMS2-F or iCMS3-F.

### 3.3. CMS and iCMS Classification of Adenomas

Five patients had CRC and synchronous adenoma. The numbers of synchronous colorectal adenomas were 1 (*n* = 2), 2 (*n* = 1), 3 (*n* = 1), and 4 (*n* = 1) (Table 1). There were nine adenomas with low-grade dysplasia, two adenomas with high-grade dysplasia, and one traditional serrated adenoma. Incidentally, the most abundant CMSs from the CiberSortx and iCMS results were CMS3 and iCMS3 for all five adenocarcinomas. Interestingly, the most abundant CMSs and iCMSs for the matched adenomas were CMS3 and iCMS3, implying that the CMS cell types observed in adenocarcinomas and adenomas are more or less patient-specific.

### 3.4. CRC Characteristics and Association with CMS Classification

Among 29 CRCs, the most common initial tumor node metastasis (TNM) stages were II (*n* = 13) and III (*n* = 11), followed by IV (*n* = 4) and I (*n* = 1) (Table 1). There was no association between the CMS and CRC stage, either by RF or iCMS (Table 2). However, among the four cases with initial stage IV metastasis, most (*n* = 3, 75%) were CMS2/iCMS2. If metastasis occurred in the three cases during the mean three-year follow-up, CMS2/iCMS2, CMS4/iCMS2, and CMS4/iCMS3 were three, one, and three cases, respectively.

CRC was located in the left colon in 21 cases and the right colon in 8 cases, and there was no association between RF and iCMS classification.

All colorectal adenocarcinoma tissues were tested for MSI status. Four cases showed MSI type, and all CMS1 cases had RF classification. Most patients (*n* = 3) were classified as iCMS3-MSI.

### 3.5. Variant Calling and Pathways

Somatic mutation profiles were summarized using canonical CRC pathways (Supplementary Figure S1). In the MAPK and TGFB pathways, nonsynonymous and frameshift mutations were observed, whereas nonsense mutations were mainly found in APC genes in the WNT pathway. However, no clear association was observed between MSI status and tissue type. Regarding TP53 mutations, none of the 11 adenomas had nonsynonymous mutations, whereas four out of five matched adenocarcinomas had nonsynonymous mutations.

## 4. Discussion

Here, we report a bulk transcriptomic study on CRC and synchronous colorectal adenomas using RNA-seq. Using the traditional CMS scheme with an RF tool, approximately half of the samples were classified as CMS4. However, after inferring bulk data using CiberSortx, approximately half of the adenocarcinoma samples had approximately 50% or more CMS2 cell types, whereas in the other adenocarcinoma and adenoma samples, CMS3 was the major type. In the five patients who had colorectal adenocarcinoma with synchronous colorectal adenomas, the CMS from CiberSortx was CMS3 in all adenocarcinoma and adenoma samples. Our CiberSortx results are consistent with the revised iCMS classification [3].

In 2015, based on gene expression profiles from bulk tumors, an international consortium identified heterogeneity of CRC by CMS classification [2], which represented four distinct subtypes (CMS 1–4) [12,13]. However, bulk transcriptomes measure total gene expression in heterogeneous tissues; hence, the transcriptomes of component cells, their proportions, and tumor microenvironment interactions are obscured. With the recent advent of single-cell-level transcriptomics, scRNA-seq characterizes transcriptomes at a cellular resolution, identifying cell types and their expression profiles. Recently, Joanito et al. reexamined several CRC scRNA-seq datasets and revised their CMS classification system [3]. When the bulk transcriptome datasets were reclassified, most tumors classified as CMS2 or CMS3 remained intact as iCMS2 or iCMS3, respectively. Conversely, 97% of tumors classified as CMS1 were reclassified as an iCMS3 subset with MSI. Moreover, CMS4 tumors have been reclassified as microsatellite-stable iCMS2 or iCMS3 tumors. We used CiberSortx to determine a cell-type signature matrix generated from a CRC scRNA-seq dataset. The global cell proportions observed in our samples resembled those observed in Korean scRNA-seq datasets [14]. Additionally, the CMSs from CiberSortx did not match the traditional CMS scheme using the RF tool, but they were consistent with the iCMS classification. Joanito et al. argued that CMS4 samples should be reassigned to the fibrosis subtypes of iCMS2 or iCMS3. We addressed this issue using myofibroblast abundance based on our CiberSortx results. RF classified approximately half of the CRC samples as CMS4, whereas CMS4 was hardly detected in the CiberSortx results, and all these samples were clustered with iCMS2 or iCMS3. Because they were enriched with myofibroblasts according to the CiberSortx classification, we tentatively designated them as iCMS2-F or iCMS3-F.

We focused on the CMS classification associated with adenoma because CRC arises via a stepwise progression from normal colon epithelial tissues to adenomas and then to CRC. Such a transition from traditional tubular adenomas to adenocarcinomas occurs over more than 10–15 years and is accompanied by sequential changes in the Wnt signaling pathway followed by the RAS-RAF-MAPK, TGF-b, and PI3K-AKT pathways [15]. Our adenoma specimens were from patients with CRC; adenomas in patients with malignancy, as opposed to those without malignancy, might have a greater potential to differentiate into adenocarcinomas. Interestingly, CMSs and iCMSs for adenocarcinomas and synchronous adenomas were CMS3 from CiberSortx and iCMS3, respectively. This result is comparable to that of a previous study [16], which collected triplicate tissue samples (primary CRC, adjacent normal tissue, and adenoma; *n* = 15) from five patients. The CMS classifier results showed that all 11 adenomas belonged to CMS3 and developed into different carcinoma CMSs such as CMS 2/3/4 by RF analysis. However, the CMS and iCMS adenocarcinomas and synchronous adenomas were all CMS3 from CiberSortx and iCMS3. Thus, CiberSortx and iCMS analyses, which are based on a single-cell transcriptome database, can be more reliable for classifying cancer and precancerous lesions. To investigate whether cumulative mutations and genetic alterations during the development of CRC affect this proposed classification and CRC typing, we examined five matched pairs of adenomas and adenocarcinomas regarding TP53 mutations. While none of these five adenomas had nonsynonymous mutations, four carcinomas had nonsynonymous mutations in TP53. Regarding the molecular classification, we reported that CMS cell types are more or less patient-specific. Putting these together, we have not found any cases of class switchover cancer development. However, our cases are limited at this time to give a definite answer.

Concerning CMS and its clinicopathological characteristics, the original work that reported RF classification models characterized CMS1 as frequently observed in samples showing MSI. Our samples, which were classified as CMS1, showed an MSI phenotype. However, in the iCMS classification, most MSI-H tumors were classified as iCMS3 (iCMS3-MSI type), which is consistent with previous results [3]. Although a previous study reported that right-sided tumors were mainly iCMS3 (66%) and left-sided tumors were mainly iCMS2 (68%), there was no relationship between tumor location and CMS classification in our data.

Poor relapse-free survival is a feature of the CMS4/iCMS3-F subtype, which shows the lowest overall survival, followed by the CMS4/iCMS2-F subtype [3]. Here, among the seven metastasis cases, three new cases of metastasis were observed during follow-up, two were CMS4/iCMS3-F and one was CMS4/iCMS2-F. The TNM stage was IIIB at diagnosis in them. Thus, recurrence cannot be accurately predicted solely based on the TNM staging system [17]. As CRC is a heterogeneous disease, a comprehensive molecular classification system needs to be established to stratify patients with CRC per the target gene expression profiles; characterizing their distinct immunology could predict recurrence or metastasis and enable the prioritization of customized treatment.

Previous studies analyzing oncogene cumulative mutation and activation, tumor suppressor gene inactivation, and gene deletion associated with CRC carcinogenesis have provided a partial interpretation of genetic alterations that occur during CRC development [18,19,20,21]. Analyzing the transcriptome profiles of the mucosal adenoma–carcinoma sequence in CRC may shed light on mechanisms underlying CRC onset. Here, all adenoma samples were classified as CMS3, which is consistent with the previous results [2]. CMS3 adenomas may develop into tumors of the same CMS, implying that the molecular characteristics of CRC are similar to those of synchronous adenomas. RNA-seq data obtained from patient-matched samples of the adenoma–carcinoma sequence in CRC, as performed here, can reduce the effects of differences among patients.

The strength of our study is that the traditional classification relying on random forest machine learning has been based on molecular signatures that had been developed from bulk RNA-seq data. The drawback of that system is that it ignores cellular heterogeneity. As we and others have shown, such a classification is often contradictory to those obtained from scRNA-seq. We use molecular signatures derived from scRNA-seq data. As such, our results are congruent with the state-of-the-art molecular classification based on scRNA-seq. However, there are also several limitations. Recently, a pre-cancer atlas study identified two cell types, one attributable to adenomatous polyps and the other to sessile serrated lesions (SSLs). Most adenomatous polyp markers showed higher expression in iCMS2 patients, whereas SSL markers were upregulated in iCMS3 [3]; however, there was only one sample with traditional serrated adenoma, which showed higher expression in iCMS3, along with other adenomatous polyps. Thus, the molecular characteristics according to pre-cancer cell type need to be studied using more samples. Second, because an in silico analysis was performed to analyze cellular heterogeneity, there may have been differences in the experimental results. To minimize such differences, we applied the S-mode using scRNA-seq of Korean CRC and normal samples.

## 5. Conclusions

In conclusion, the new iCMS classification, coupled with the application of an in silico cell fractionation method, can provide a harmonious molecular dissection of complex diseases such as CRC.

## Figures and Tables

**Table 1:** Clinical characteristics and CMS classification of colorectal cancer and adenoma.

Sample	Gender	Age	CMS1	CMS2	CMS3	CMS4	RF Nearest	iCMS	MSI Status	Differentiation	Initial TNM Stage		Final Metastasis	Location	Size	Adenoma	Location	Size
SMK.130.1.TR	F	53	0.05%	93.51%	5.86%	0.58%	2	2	MSS	MD	pT3N0M0	IIA	M0	SC	4.2			
SMK.197.1.TR	M	59	0.00%	71.25%	28.53%	0.23%	4	2	MSS	MD	pT3N0M0	IIA	M0	SC	4.9			
SMK.195.1.TR	M	50	0.00%	69.85%	30.06%	0.09%	4	2	MSS	MD	pT3N0M0	IIA	M0	REC	7			
SMK.126.1.TR	M	54	0.00%	68.70%	31.18%	0.12%	2	2	MSS	WD	T4bN3M1	IVA	M1	RSJ	8.3			
SMK.104.1.TR	M	57	0.00%	67.65%	32.35%	0.00%	4	2	MSS	MD	pT3N1cM0	IIIB	M0	AC	5.0			
SMK.087.1.TR	M	60	0.00%	67.33%	32.67%	0.00%	4	2	MSS	MD	pT3N0M0	IIA	M0	RSJ	11.7			
SMK.081.1.TR	F	76	0.00%	65.96%	33.67%	0.37%	2	2	MSS	MD	pT3N2aM0	IIIB	M0	AC	5.3			
SMK.099.1.TR	F	55	0.00%	65.83%	33.93%	0.24%	2	2	MSS	MD	pT4aN2bM1	IVB	M1	SC				
SMK.117.1.TR	M	42	0.00%	64.92%	34.86%	0.22%	4	2	MSS	MD	pT3N1bM1	IVB	M1	RSJ	5.3			
SMK.236.1.TR	M	64	0.00%	60.90%	38.59%	0.51%	4	2	MSS	MD	pT3N0M0	IIA	M0	SC				
SMK.114.1.TR	F	75	0.00%	60.19%	39.74%	0.06%	4	2	MSS	MD	pT3N0M0	IIA	M0	AC	4.3			
SMK.216.1.TR	F	63	16.23%	56.75%	27.02%	0.00%	1	2	MSI	PD	pT3N0M0	IIA	M0	AC				
SMK.185.1.TR	F	51	0.00%	51.18%	48.70%	0.12%	2	2	MSS	WD	T3N2aM0	IIIB	M1	REC	4.2			
SMK.111.1.TR	M	68	0.00%	45.54%	54.46%	0.00%	4	2	MSS	MD	pT3N1aM0	IIIB	M0	SC	3.8			
SMK.084.1.TR	M	54	0.00%	36.88%	62.75%	0.36%	4	3	MSS	MD	pT3N1aM0	IIIB	M1	TC	3.7			
SMK.206.1.TR	M	67	0.00%	29.07%	70.69%	0.23%	2	3	MSS	MD	pT3N1bM0	IIIB	M0	SC				
SMK.176.1.TR	M	66	0.00%	27.38%	72.62%	0.00%	4	3	MSS	WD	pT3N0M0	IIA	M0	SC				
SMK.176.4.TR(A)	M	66	0.00%	7.09%	92.74%	0.17%	3	3						REC	1	TA, LGD	REC	1.0
SMK.199.1.TR	M	58	0.00%	26.03%	73.94%	0.04%	2	3	MSS	MD	T3N1M0	IIIB	M0	REC	5.5			
SMK.205.1.TR	M	60	0.00%	20.06%	79.94%	0.00%	4	3	MSS	MD	pT3N2aM0	IIIB	M0	REC				
SMK.222.1.TR	M	61	8.92%	19.49%	71.60%	0.00%	1	3	MSI	Mucinous	pT3N0M0	IIA	M0	AC	7.0			
SMK.101.1.TR	F	64	0.00%	18.99%	80.89%	0.12%	4	3	MSS	MD	pT4aN1bM1	IVA	M1	RSJ	4.8			
SMK.116.1.TR	M	58	0.00%	17.78%	82.22%	0.00%	4	3	MSS	MD	pT4aN1aM0	IIIC	M1	SC				
SMK.137.1.TR	M	60	0.00%	17.13%	82.66%	0.21%	3	3	MSS	MD	pT3N1cM0	IIIB	NA	REC	8.5			
SMK.186.4.TR	M	54	0.00%	15.87%	84.05%	0.08%	2	3	MSS	MD	pT3N0M0	IIA	M0	SC				
SMK.186.2.TR(A)	M	54	0.00%	6.29%	93.67%	0.04%	3	3						HF	1	TA, LGD	TC	1.0
SMK.186.3.TR(A)	M	54	0.00%	5.02%	94.52%	0.45%	3	3						DC	0.7	TA, LGD	DC	0.8
SMK.198.1.TR	M	83	2.57%	13.38%	83.97%	0.08%	1	3	MSI	MD, mucinous	pT3N1bM0	IIIB	M0	SC	4.6			
SMK.106.1.TR	F	53	0.00%	11.81%	88.15%	0.04%	2	3	MSS	MD	pT3N0M0	IIA	M0	RSJ	2.5			
SMK.083.1.TR	M	79	0.00%	9.81%	90.12%	0.07%	3	3	MSS	MD	pT2N0M0	I	M0	TC	2.0			
SMK.083.5.TR(A)	M	79	0.00%	46.77%	52.91%	0.32%	3	3						REC	2	TA, HGD	REC	2.0
SMK.191.1.TR	M	77	2.50%	0.83%	96.67%	0.00%	1	3	MSI	MD	pT3N0M0	IIA	M0	TC				
SMK.191.2.TR(A)	M	77	7.13%	5.00%	87.57%	0.30%	4	3						TC	0.7	TA, LGD	TC	0.7
SMK.191.3.TR(A)	M	77	0.40%	0.00%	99.45%	0.16%	3	3						TC	0.7	TSA, LGD	TC	0.7
SMK.191.4.TR(A)	M	77	0.00%	0.00%	100%	0.00%	3	3						SC	1	TA, LGD	SC	1.0
SMK.082.8.TR	M	87	0.00%	0.00%	100%	0.00%	4	3	MSS	MD	pT4aN0	IIB	NA	SC	4.0			
SMK.082.2.TR(A)	M	87	0.00%	16.83%	83.00%	0.18%	3	3						TC	1.5	TA, LGD	TC	1.5
SMK.082.3.TR(A)	M	87	0.00%	0.00%	99.92%	0.08%	4	3						TC	0.8	TA, LGD	TC	0.8
SMK.082.6.TR(A)	M	87	0.00%	17.44%	82.33%	0.22%	3	3						DC	2	TA, HGD	DC	2.0

F, female, M, male, MSI, microsatellite instability, MSS, microsatellite stable, MD, moderate differentiate, PD, poor differentiate, AC, ascending colon, TC, transverse colon, HF, hepatic flexure, DC, descending colon, SC, sigmoid colon, RSJ, rectosigmoid junction, REC, rectum.

**Table 2:** Association of CMS classification and CRC characteristics.

	Stage		Location	
<RF>	I, II (*n* = 14)	III, IV (*n* = 15)	Left (*n* = 21)	Right (*n* = 8)
CMS 1	3 (21.4%)	1 (6.7%)	CMS 1	3 (21.4%)
CMS 2	3 (21.4%)	6 (40.0%)	CMS 2	3 (21.4%)
CMS 3	1 (7.1%)	1 (6.7%)	CMS 3	1 (7.1%)
CMS 4	7 (50%)	7 (46.7%)	CMS 4	7 (50%)
<iCMS>			<iCMS>	
iCMS 2	7 (50%)	7 (46.7%)	iCMS 2	7 (50%)
iCMS 3	7 (50%)	8 (53.3%)	iCMS 3	7 (50%)

RF, random forest.

## Data Availability

The most data presented in this study are available in this article (and Supplementary Materials) and other data can be shared up on request.

## Supplementary Materials

The following supporting information can be downloaded at: https://www.mdpi.com/article/10.3390/cancers15194851/s1, Figure S1: Oncoprint of CRC.
