# Supplementary figures and images for: RNA-Seq-Based Molecular Classification Analyses in Colorectal Cancer and Synchronous Adenoma
Source: Cancers (Basel). 2023 Oct 4;15(19):4851. doi: 10.3390/cancers15194851 (PMC10571664; doi:10.3390/cancers15194851)

Oncoprint of CRC

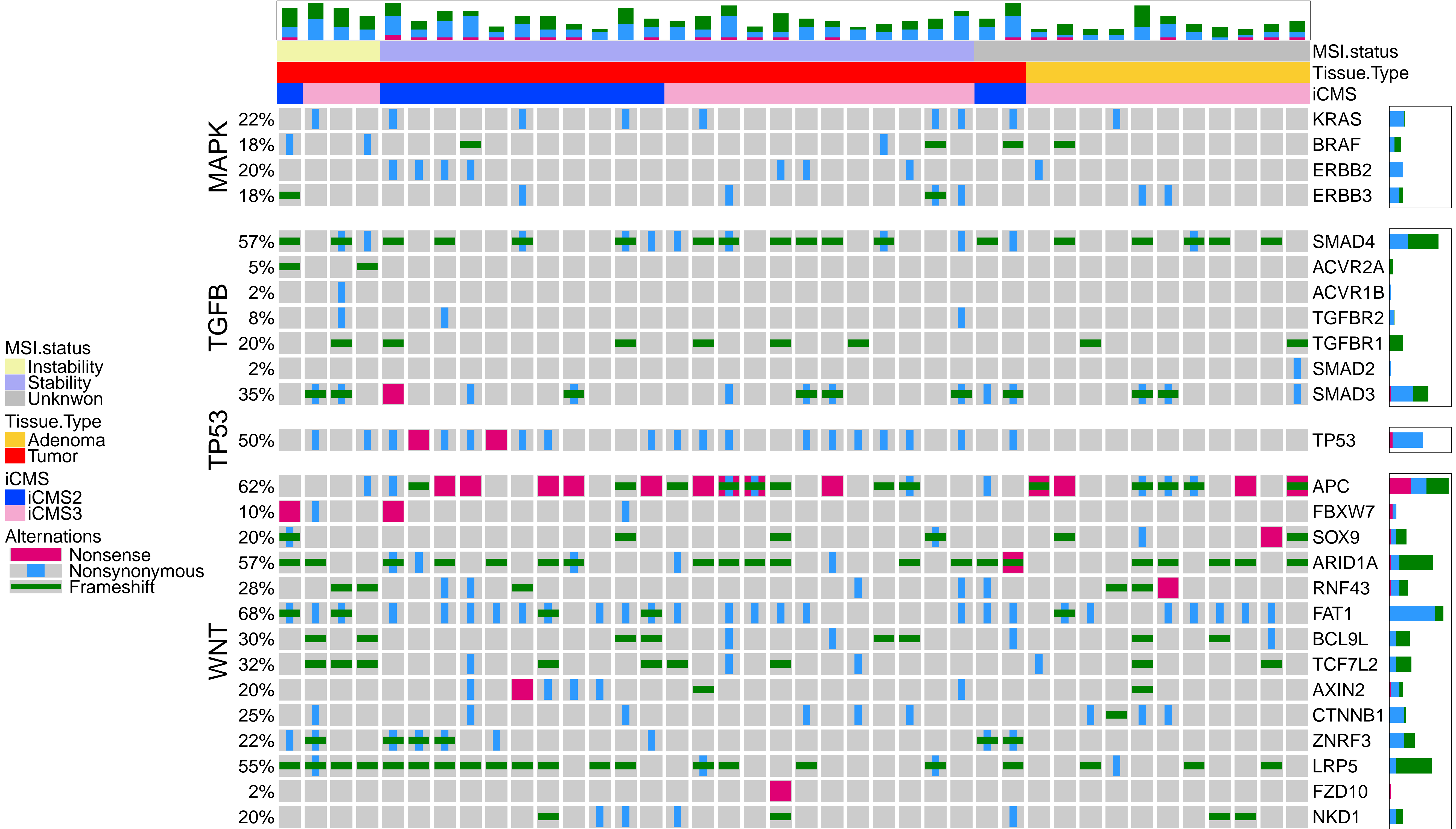

Supplement: Supplementary file 1 [file cancers-15-04851-s001.zip › cancers-2600443-supplementary.pdf]
